# A *Taq*Man Probe-Based Multiplex Real-Time PCR for Simultaneous Detection of Porcine Epidemic Diarrhea Virus Subtypes G1 and G2, and Porcine Rotavirus Groups A and C

**DOI:** 10.3390/v14081819

**Published:** 2022-08-19

**Authors:** Letian Zhang, Zhiwen Jiang, Zitong Zhou, Jiumeng Sun, Shiyu Yan, Wenting Gao, Yuekun Shao, Yuhe Bai, Yifan Wu, Zefei Yan, Shouzhi Sheng, Alexander Lai, Shuo Su

**Affiliations:** 1 Jiangsu Engineering Laboratory of Animal Immunology, Institute of Immunology, College of Veterinary Medicine, Nanjing Agricultural University, Nanjing 210095, China; 2 School of Science, Technology, Engineering, and Mathematics, Kentucky State University, Frankfort, KY 40601, USA

**Keywords:** real-time PCR, detection, PEDV G1, PEDV G2, RVA, RVC

## Abstract

Porcine viral diarrhea diseases affect the swine industry, resulting in significant economic losses. Porcine epidemic diarrhea virus (PEDV) genotypes G1 and G2, and groups A and C of the porcine rotavirus, are major etiological agents of severe gastroenteritis and profuse diarrhea, particularly among piglets, with mortality rates of up to 100%. Based on the high prevalence rate and frequent co-infection of PEDV, RVA, and RVC, close monitoring is necessary to avoid greater economic losses. We have developed a multiplex *Taq*Man probe-based real-time PCR for the rapid simultaneous detection and differentiation of PEDV subtypes G1 and G2, RVA, and RVC. This test is highly sensitive, as the detection limits were 20 and 100 copies/μL for the G1 and G2 subtypes of PEDV, respectively, and 50 copies/μL for RVA and RVC, respectively. Eighty-eight swine clinical samples were used to evaluate this new test. The results were 100% in concordance with the standard methods. Since reassortment between porcine and human rotaviruses has been reported, this multiplex test not only provides a basis for the management of swine diarrheal viruses, but also has the potential to impact public health as well.

## 1. Introduction

Viral diarrheal disease due to coronaviruses and rotaviruses (RVs) is currently a serious threat to the swine industry, resulting in significant economic losses worldwide. Porcine epidemic diarrhea virus (PEDV), porcine group A rotavirus (RVA), and group C rotavirus (RVC) are the most common pathogens among newborn piglets, with a mortality rate of up to 100%. However, their correct diagnosis is problematic due to their similar clinical symptoms, including vomiting and profuse diarrhea. The recent emergence of PEDV group 2 (G2) and re-emergence of G1 are particular of concern, as they are highly pathogenic for suckling piglets [1]. As a coronavirus with frequent recombination and mutations [2], several genotypes of PEDV, based on the spike (S) gene, have emerged and been identified as G1a, G1b, G2a, and G2b [3,4]. The emergence of novel recombinants or mutated PEDV strains may result with a higher virulence in pigs [5] and reduce existing vaccine efficacy [6,7]. Currently, multiple genotypes of the PEDV strains are co-circulating in the pig population [8]. A test that provides rapid detection and differentiation of the PEDV strains is urgently needed to prevent epizootics of these viruses.

Rotavirus, on the other hand, causes mild to severe diarrhea for animals and humans [9]. Rotaviruses are classified into 9 groups or species (RVA-RVD and RVF-RVJ) [10,11], based on the amino acid sequence of the structural protein, VP6 [12]. Four RVs groups, including RVA, RVB, RVC, and RVH, are pathogenic to both humans and pigs [13]. RVA is the main rotavirus group, with multiple genotypes that cause porcine diarrhea. Notably, reassortment between human and porcine rotavirus strains has been reported [14,15]; hence, it poses a public health threat [16]. Like RVA, RVC also has the ability to infect a variety of animals, and particularly, it has a high prevalence rate among newborn piglets. Recent studies have shown that several genes (e.g., VP6 or nsP4) of the porcine RVC strains are homologues to human RVC strains, supporting its zoonotic potential [17,18]. In addition to a high prevalence rate of RVC among pigs, co-infection with RVA occurs frequently [19]. However, the extent of the RVC infection or co-infection with RVA is unknown. Therefore, there is a need to include these two rotaviruses in the etiological screening of porcine viral diarrheal diseases [9,20]. Moreover, with the frequent interspecies transmission by coronaviruses and rotaviruses, the threat of these viruses to public health and livestock production is significant [21,22]. Therefore, a means of rapid detection and a differentiating diagnostic test will assist in the implementation of effective mitigation strategies.

While PEDV, RVA, and RVC can be diagnosed by individual PCR testing, the interpretation of mixed viral infections is problematic, and sometimes results in confusion. To implement an effective monitoring of these viruses, a rapid, accurate, and differential diagnostic tool is needed. We describe a multiplex real-time PCR assay that can effectively detect, identify, and differentiate these viruses. 

## 2. Materials and Methods

### 2.1. Design of the Primers and Probes

Multiple sequence alignments were carried out using all available sequences of the S gene from the G1 and G2 genotypes of PEDV, and the VP6 gene from RVA and RVC in GenBank (as of 24 October 2021). We used MEGA7 software to confirm the highly conserved regions within the two genotypes of PEDV as well as within two groups of RVs. Oligo (Version 7.60) software was used to design four pairs of primers and four corresponding probes. In order to ensure the fluorescence signals in this multiplex system do not interfere with one another, four fluorescent dyes with large differences in wavelength were selected for the probes. FAM, Texas Red, CY5, and VIC were used to modify the probes for PEDV-G1, PEDV-G2, RVA, and RVC, respectively. The primers and probes sequences are shown in Table 1. These primers and hydrolysis probes were synthesized by Sangon Biotech (Shanghai, China) Co., Ltd.

### 2.2. Development and Optimization of the Multiplex Real-Time PCR

Viral RNA from the positive samples for these four target pathogens was extracted using TRIzol Reagent (Vazyme, Nanjing, China), followed by using HiScript III RT SuperMix for qPCR Kit (Vazyme, Nanjing, China) to synthesize the first-strand cDNA. To generate plasmids that contain the target genes, specific cloning primers were amplified with *Taq* polymerase, followed by cloning into a pMD18-T vector (Takara, Beijing, China). These positive plasmids were verified by sequencing, and used as standard positive controls, and for establishing standard curves for the quantitative analysis. 

Purified plasmids were quantified by OD260 (Thermo Scientific NanoDrop2000 spectrophotometers). Concentration of these plasmids were expressed as the number of copies per μL, using the following formula: y (copies/µL) = (6.02 × 10^23^) × (x (ng/µL) × 10^−9^ DNA)/(DNA length × 660) [23]. Final concentration of the plasmids was adjusted to 10^7^ copies/μL, and 10-fold serially diluted with nuclease-free water. Standard curves were established using 10-fold serial diluted plasmids, and the R^2^ (correlation coefficient) values were calculated.

We optimized the primer and probe concentrations as previously reported [24]. Final volume of the real-time PCR reaction was 20 μL, with the following ingredients: 0.3 μL of each primer (10 μM), 0.2 μL of each *Taq*Man probe (10 μM), 1 μL of template, 10 μL of 2 × AceQ qPCR Probe Master Mix (Vazyme, Nanjing, China), and the remaining volume with nuclease-free water. We carried out the amplification on Roche LightCycler^®^ 96 Instrument (Roche Life Science, Basel, Switzerland), with the following program: 95 °C for 600 s, following by 45 cycles of 95 °C for 10 s, 59 °C for 10 s, and 72 °C for 20 s. Fluorescence signals were detected at the end of each cycle of the extension step.

The sensitivity of this multiplex real-time PCR was evaluated using 10-fold serially diluted standard plasmids, ranging from 10^0^ to 10^7^ copies/µL as templates. With respect to the Ct value of detection, the lowest concentration of the standard plasmids which could be detected reproducibly was regarded as the presumed limit of detection (LOD). The limit of detection (LOD) was fine-tuned by testing the standard plasmids at the concentration of the presumed LOD and its plus or minus two-serial dilutions. The tests were repeated. 

The viruses used for testing the specificity included those that could cause respiratory and digestive tract diseases in pigs. Specimens positive for the following pathogens were used: PEDV genotype G1, PEDV genotype G2, RVA, RVC, porcine reproductive and respiratory syndrome virus (PRRSV), porcine delta coronavirus (PDCoV), transmissible gastroenteritis virus (TGEV), classical swine fever virus (CSFV), and swine influenza virus (SIV). Clinical samples were collected from affected pig farms in the Henan, Jiangsu, Anhui, and Guangdong provinces in China, and these samples were preserved at −80 °C in our laboratory. All positive samples were identified by conventional PCR in our laboratory and confirmed through DNA sequencing by Sangon Biotech (Shanghai) Co., Ltd. (Shanghai, China) Viral RNA of the positive samples was extracted using TRIzol Reagent (Vazyme, Nanjing, China), and the cDNAs of these viruses were synthesized as target templates.

Evaluation of this multiplex real-time PCR was conducted using 10-fold serially diluted standard plasmids with concentrations ranging from 1 × 10^7^ copies/μL to the LOD, in triplicate, and the experiment repeated with interval of one week between two adjacent trials. The reproducibility and the coefficient of variation (%CV) of the Ct values for different concentrations of the samples from three tests was calculated.

### 2.3. Verification of the Multiplex Real-Time PCR

Mixed infections were often misdiagnosed, as the pathogens presented at a lower concentration were not detected. To simulate mixed infections, plasmid standards from two, three, or four pathogens, at their LOD concentrations, were mixed as templates for the test. 

Finally, eighty-eight clinical samples were used for diagnostic evaluation. These samples included fecal and anal swab samples from infected pigs. To validate, all samples were further identified by conventional PCR and sequencing. Clinical performances of this multiplex real-time PCR were evaluated, as shown below.

## 3. Results

### 3.1. Evaluation of Sensitivity and Cut-Off Values

**Figure 1:**
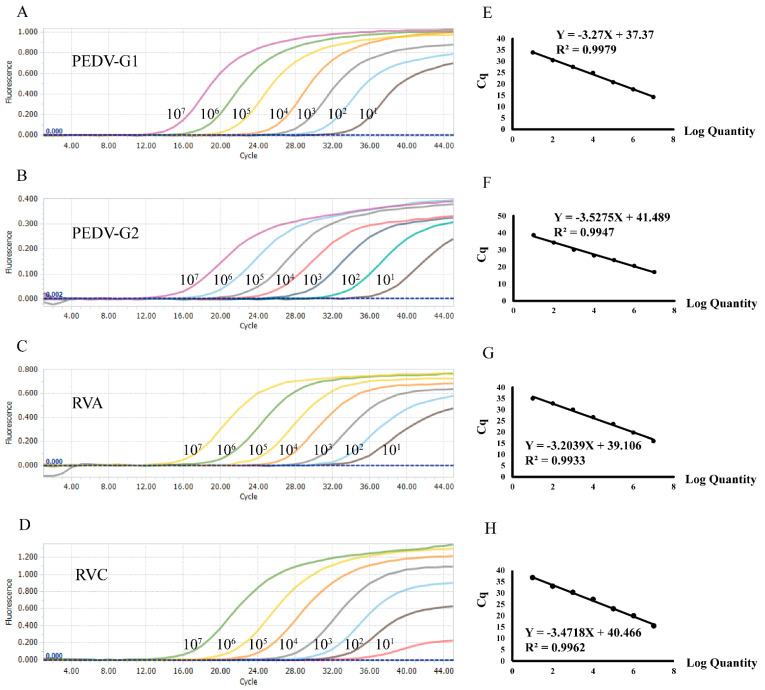
Preparation of the plasmid standards. (**A**–**D**) amplification curves (X-axis: cycle, Y-axis: fluorescence) of PEDV genotypes G1 and G2, and porcine groups A and C of the rotavirus for each plasmid standard with concentrations from 1 × 10^7^ copies/μL to 1 × 10^1^ copies/μL. (**E**–**H**) standard curves of the plasmid standards of PEDV genotypes G1 and G2, and porcine groups A and C of the rotavirus.

To establish the standard curve, concentrations of the recombinant plasmids were established, ranging from 1 × 10^7^ copies/μL to 1 × 10^1^ copies/μL through tenfold serial dilutions. The plasmid standard for each pathogen was selected for single-plex real-time PCR. Amplification efficiencies and correlation coefficients were in good linearity, with PEDV-G1 R^2^ = 0.9979, PEDV-G2 R^2^ = 0.9947, RVA R^2^ = 0.9933, and RVC R^2^ = 0.9962, respectively (Figure 1).

To determine the sensitivity, tenfold serial dilutions of linearized plasmids were added to the amplification system. The results of the sensitivity evaluation are shown in Table 2. The detection limit was 20 and 100 copies/μL for the G1 and G2 subtypes of PEDV, respectively. Whereas the detection limit was 50 copies/μL for both RVA and RVC. The Ct value was around 32–34 when the samples were at LOD concentrations. Results became unreliable when the Ct value was higher than 35. Therefore, we set Ct value at 35 as the positive cut-off, i.e., positive when Ct < 35, and negative when Ct ≥ 35.

### 3.2. Evaluation of the Specificity and Reproducibility

**Figure 2:**
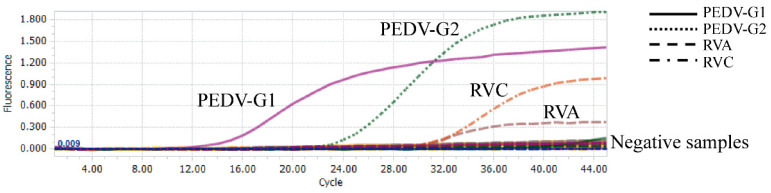
Analytical specificity of the multiplex real-time PCR. Four amplification curves (X-axis: cycle, Y-axis: fluorescence) representing samples positive for PEDV genotypes G1 and G2, and porcine group A and C rotaviruses. Negative samples included TGEV, PRRSV, CSFV, SIV, PDCoV, and the negative control.

To evaluate the specificity of this multiplex real-time PCR, cDNA from five other swine viruses, including PRRSV, PDCoV, CSFV, TGEV, and SIV, were used as templates. These viruses are common pathogens among pigs. The results are shown in Table 3. This multiplex real-time PCR assay did not amplify any of the other pathogens, hence a high specificity for the intended target viruses (PEDV subtypes G1 and G2, and for RVA and RVC, Figure 2). There was no cross-reactivity with the above five other swine viruses.

The reproducibility was evaluated by detecting the standard plasmids at concentrations ranging from 1 × 10^7^ copies/μL to the LOD. The inter-assay variability was calculated using the values obtained from different runs. The percentage coefficients of the variation (%CV) of Ct values were ranging from 0.12% to 2.84% (Table S1). The results indicated that this *Taq*Man probe-based multiplex real-time PCR is highly reproducible and reliable. 

### 3.3. Evaluation of the Simulated Co-Infections

**Figure 3:**
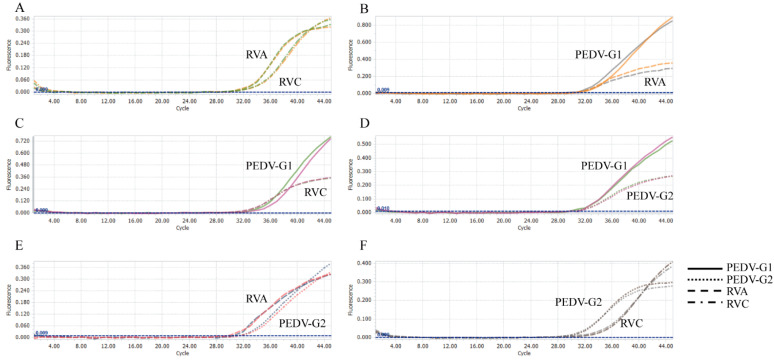
Co-infection simulation experiments with two pathogens. (**A**) amplification curves (X-axis: cycle, Y-axis: fluorescence) of RVA and RVC at concentrations of the LOD. (**B**) amplification curves (X-axis: cycle, Y-axis: fluorescence) of PEDV-G1 and RVA at concentrations of the LOD. (**C**) amplification curves (X-axis: cycle, Y-axis: fluorescence) of PEDV-G1 and RVC at concentrations of the LOD. (**D**) amplification curves (X-axis: cycle, Y-axis: fluorescence) of PEDV-G1 and PEDV-G2 at concentrations of the LOD. (**E**) amplification curves (X-axis: cycle, Y-axis: fluorescence) of PEDV-G2 and RVA at concentrations of the LOD. (**F**) amplification curves (X-axis: cycle, Y-axis: fluorescence) of PEDV-G2 and RVC at concentrations of the LOD.

**Figure 4:**
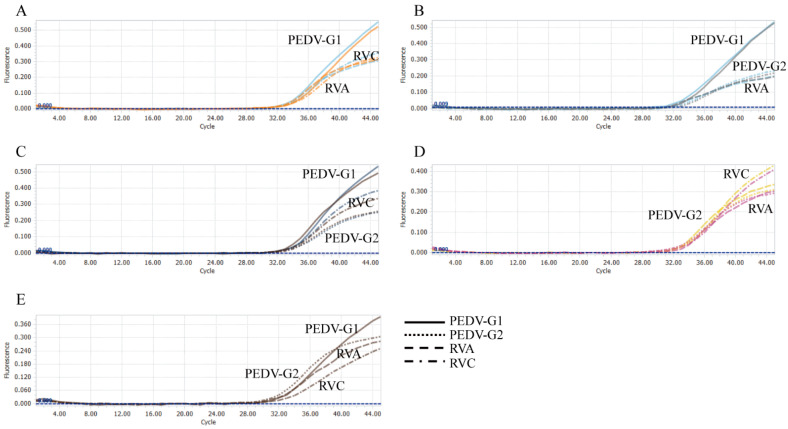
Co-infection simulation experiments with three and four pathogens. (**A**) amplification curves (X-axis: cycle, Y-axis: fluorescence) of PEDV-G1, RVA, and RVC at concentrations of the LOD. (**B**) amplification curves (X-axis: cycle, Y-axis: fluorescence) of PEDV-G1, PEDV-G2, and RVA at concentrations of the LOD. (**C**) amplification curves (X-axis: cycle, Y-axis: fluorescence) of PEDV-G1, PEDV-G2, and RVC at concentrations of the LOD. (**D**) amplification curves (X-axis: cycle, Y-axis: fluorescence) of PEDV-G2, RVA, and RVC at concentrations of the LOD. (**E**) amplification curves (X-axis: cycle, Y-axis: fluorescence) of PEDV-G1, PEDV-G2, RVA, and RVC at concentrations of the LOD.

All plasmid standards with concentrations at LOD were used to conduct a co-infection simulation experiment. This multiplex method could detect duplex, triplex, or quadruplex co-infection simulation (Figure 3 and Figure 4). Therefore, this test could detect each virus, even at the LOD concentration, from a mixed infection sample.

### 3.4. Evaluation Using the Clinical Samples

Eighty-eight (n = 88) clinical samples, including fecal and anal swab samples, were detected using this multiplex real-time PCR method. The results were 3.4% (3/88) positive for the G1 genotype of PEDV, 9.1% (8/88) for the G2 genotype of PEDV, 12.5% (11/88) for RVA, and 11.4% (10/88) for RVC. All of these viruses were detectable from the samples. The positive rate of G2 genotype of PEDV was higher than that of G1 genotype of PEDV. This is consistent with the fact that G2 genotype of PEDV has become the most prevalent genotype in China. Notably, the positive rate for RVC was similar to that of RVA, indicating that the prevalence rate of this virus had been previously underestimated. It is worth noting that there were some other pathogens among these clinical samples. There were no false positive results, therefore, validating the specificity of this test. These samples were further tested by conventional PCR and sequencing. There was a 100% concordance.

We further evaluate co-infections from these positive clinical samples. One sample had PEDV G1 and RVC, five samples had PEDV G2 and RVA, three samples had RVA and RVC, and, significantly, one sample had triple infections: PEDV G2, RVA, and RVC (Table S2). Therefore, occurrence of mixed viral diarrhea had been significantly underestimated among these pig farms.

## 4. Discussion

The high prevalence rates of multiple genotypes of PEDV and RV in pigs impedes the prevention and control of porcine viral diarrheal diseases [25,26]. The G2 genotype of PEDV has emerged as the main genotype circulating in the swine population since 2010. In addition, an emerging novel genotype of PEDV has been found, thus increasing the complexity of PEDV epizootics [27]. A rapid identification of the G1 and G2 subtypes of PEDV allows evidence-based development of homotypic vaccines for the prevention and control of these viral infections. Furthermore, an effective control reduces the risk of recombination among these PEDV strains. Rotavirus is also associated with piglet diarrhea, and mixed infection with other porcine enteroviruses is common [28]. A timely and correct diagnosis of these virus is essential for mitigation and treatment, when outbreaks or epizootics of these swine viral diarrheal disease occur [29,30]. In addition, this highly sensitive multiplex real-time PCR can be utilized as an early screening tool for porcine viral diarrhea disease, for epidemiology studies, and to monitor the spread of these viruses.

Of note, establishing a sensitive and specific test is difficult. Primers from the conserved regions, e.g., of the N gene or ORF1a gene, can be used for a robust detection test, but they cannot be used as a tool for genotyping, as the S gene has greater genetic diversity [24,31]. Compared to the G1 genotype of PEDV, G2 genotype exhibits unique insertions and deletions in the S gene [32], proving targets for differentiation. Our approach was, by first identifying specific primers for genotype detection and differentiation using the S genes (for G1 and G2 genotypes of PEDV), followed by designing specific primers and probes, after intensive sequence alignments of the “conserved regions” among viruses of the same genotype, for the specificity. The primers for G2 genotype of PEDV selected were conserved among G2a and G2b strains, and vice versa. From the results of evaluation of specificity, these primers and probes also differentiated genotypes 1 and 2 of PEDV. Similarly, the VP6 gene of rotavirus was selected for primers design, because this gene has been used to detect and differentiate rotavirus species [12]. However, the VP6 gene has high variabilities among RVC strains, and there is only a narrow conserved region at the 3′ end. To detect diverse RVC strains, we introduced a short region with degenerated bases in the upstream primer, to enable the detection of more diverse field strains.

There had been several duplex real-time PCR methods available to differentiate between G1 and G2 PEDV, or between RVA and RVC. Zhao et al. (2014) and Su et al. (2018) developed a duplex *Taq*Man real-time PCR that can differentiate classical and variant PEDV [33,34], but the limit of detection was approximately 5 × 10^2^ DNA copies for each virus. Marthaler et al. (2014) developed a duplex *Taq*Man real-time PCR that can differentiate between RVA and RVC [35], and had shown that positive rates of RVA (62%) and RVC (53%) from their 7508 tested samples. These above methods can efficiently differentiate between two genotypes or species of viruses, but they cannot distinguish these four types of common swine diarrheal viruses. Our multiplex real-time PCR, by using four signals (FAM, Texas Red, CY5, and VIC), it can detect and differentiate four target pathogens. Furthermore, based on the different wavelengths of these four signals, there is no interfere, and the fluorescence signal values can be detected concurrently and simultaneously in the same reaction tube. Mixing of multiple primers and probes is usually problematic, as the annealing temperatures for different primer-sets are usually different. We have effectively solved this problem by optimizing the reaction conditions. This multiplex real-time PCR is highly specific, as it can accurately detect G1 and G2 PEDV strains as well as RVA and RVC, while the other five swine viruses were not detected from simulated or clinical samples. 

Of note, specific viruses were also detected and identified from clinical samples with mixed infections (Table S2), indicating that co-infections are common in diarrheal pigs [19]. Previous studies have shown a high positive rate of RVA and RVC in diarrhea samples. Our results further support this observation. The true prevalence rates of these viruses in China had been underestimated [36]. Therefore, future epidemiological studies of porcine viral diarrhea should focus on this virus. 

In summary, we had developed a multiplex real-time PCR for the detection and differentiation of four common porcine viral diarrhea, including the PEDV subtypes G1 and G2, RVA, and RVC. This real-time PCR has a high sensitivity and specificity. Furthermore, samples from anal swabs, feces, and tissues can be used. This test reduces the detection time and cost. By providing a rapid, sensitive, and specific test for multiple swine diarrhea viruses, it will positively impact the swine industry, to prevent disruption of food supply.

## Figures and Tables

**Table 1:** Primers and probes for the multiplex assay.

Primer/Probe Name	Sequence 5′-3′	Gene	Length (bp)
PEDV-G1-F	TGTTTTGGGTGGTTATCTACCTA	S	168 ^a^
PEDV-G1-R	AGCTGGTAACCACTAGGAT		
PEDV-G1-Probe	FAM-TGTGCCACAGTACCAGCTAGAAGA-MGB		
PEDV-G2-F	CCAGTACTTTCAACACTTAGCCTA	S	195 ^b^
PEDV-G2-R	GCCACTAGCAGTTGGATG		
PEDV-G2-Probe	Texas Red-CAAGTTGAATTGACACCCTGGTTT-BHQ2		
RVA-F	CAACGAAACGGAATAGCACC	VP6	123 ^c^
RVA-R	CCGCCTATTCTGTAGATTCCAA		
RVA-Probe	CY5-ACCCGACAGCTTTCTTAGTGCTT-BHQ3		
RVC-F	GTGAAGAGAATGGTGHTGTAG	VP6	121 ^d^
RVC-R	CATGCGCATTTGCCCCTACGC		
RVC-Probe	VIC-CATGATTCACGAATGGGTTTAG-BHQ1		

The lengths of the amplified genes were determined by ^a^ GenBank accessions AF353511.1, ^b^ JX088695.1, ^c^ MF462325.1, and ^d^ AB889499.1, respectively.

**Table 2:** Sensitivity of the multiplex real-time PCR assay.

Pathogen	Concentration (Copies/μL)	Repeat Times	Positive Number	Positive Rate	95% Positive Rate
PEDV-G1	2 × 10^1^	23	23	100%	>95%
	1 × 10^1^	23	0	0%	<95%
PEDV-G2	1 × 10^2^	23	23	100%	>95%
	1 × 10^1^	23	0	0%	<95%
RVA	5 × 10^1^	23	23	100%	>95%
	1 × 10^1^	23	0	0%	<95%
RVC	5 × 10^1^	23	23	100%	>95%
	1 × 10^1^	23	0	0%	<95%

**Table 3:** Specificity of the multiplex real-time PCR assay.

Sample Type	Controls	Multiplex Real-Time PCR in This Study (Ct Value)			
		PEDV-G1	PEDV-G2	RVA	RVC
Fecal	PEDV-G1	12.57	-	-	-
Anal swab	PEDV-G2	-	21.49	-	-
Fecal	RVA	-	-	29.04	-
Anal swab	RVC	-	-	-	30.99
Anal swab	TGEV	-	-	-	-
Fecal	PDCoV	-	-	-	-
Lung	PRRSV	-	-	-	-
Lung	CSFV	-	-	-	-
Nasal swab	SIV	-	-	-	-

## Data Availability

Not applicable.

## Supplementary Materials

The following supporting information can be downloaded at: https://www.mdpi.com/article/10.3390/v14081819/s1. Table S1: Repeatability results of the duplex real-time PCR assay, Table S2: Co-infection result of clinical specimens.
