# Supplementary material for: A TaqMan Probe-Based Multiplex Real-Time PCR for Simultaneous Detection of Porcine Epidemic Diarrhea Virus Subtypes G1 and G2, and Porcine Rotavirus Groups A and C
Source: Viruses. 2022 Aug 19;14(8):1819. doi: 10.3390/v14081819 (PMC9413770; doi:10.3390/v14081819)
Supplement: Supplementary file 1 [file viruses-14-01819-s001.zip › viruses-1870580-supplementary.pdf]

A *Taq*Man Probe-Based Multiplex Real-Time PCR for Simultaneous Detection of Porcine Epidemic Diarrhea Virus Subtypes G1 and G2, and Porcine Rotavirus Groups A and C

Letian Zhang [1,†], Zhiwen Jiang [1,†], Zitong Zhou [1], Jiumeng Sun [1], Shiyu Yan [1], Wenting Gao [1], Yuekun Shao [1], Yuhe Bai [1], Yifan Wu [1], Zefei Yan [1], Shouzhi Sheng [1], Alexander Lai [2] and Shuo Su [1,*]

[1] Jiangsu Engineering Laboratory of Animal Immunology, Institute of Immunology, College of Veterinary Medicine, Nanjing Agricultural University, Nanjing 210095, China

[2] School of Science, Technology, Engineering, and Mathematics, Kentucky State University, Frankfort, KY 40601, USA

* Correspondence: shuosu@njau.edu.cn

† These authors contributed equally to this work.

Table S1. Repeatability of the duplex real-time PCR assay.

Plasmid	Concentration (copies/μL)	Intra-assay Ct value			Inter-assay Ct value	CV%
		Group1(average)	Group2(average)	Group3(average)	Mean±SD	
PEDV-G1	10^1	33.66	32.41	32.05	32.71±0.69	2.11%
	10^2	30.94	31.04	30.58	30.85±0.20	0.65%
	10^3	27.87	27.55	27.03	27.48±0.35	1.27%
	10^4	24.44	24.12	23.65	24.07±0.32	1.33%
	10^5	21.05	20.87	20.48	20.80±0.24	1.15%
	10^6	17.49	17.43	17.10	17.34±0.17	0.98%
	10^7	14.05	13.77	13.80	13.87±0.13	0.94%
PEDV-G2	10^2	34.03	34.50	34.34	34.29±0.20	0.58%
	10^3	31.33	32.08	31.04	31.48±0.44	1.40%
	10^4	28.29	28.80	28.12	28.40±0.29	1.02%
	10^5	24.73	25.51	25.11	25.12±0.32	1.27%
	10^6	21.37	22.06	21.38	21.60±0.32	1.48%
	10^7	18.34	18.10	17.63	18.02±0.29	1.61%
RVA	5×10^1	33.09	33.13	33.35	33.19±0.12	0.36%
	10^2	32.82	32.93	32.91	32.89±0.05	0.15%
	10^3	29.23	30.06	29.98	29.76±0.37	1.24%
	10^4	26.38	26.89	26.83	26.70±0.23	0.86%
	10^5	22.97	23.45	23.49	23.30±0.24	1.03%
	10^6	19.68	20.90	19.92	20.17±0.53	2.63%
	10^7	16.26	17.42	16.94	16.88±0.48	2.84%

RVC	5×10^1	34.21	34.31	34.27	34.26±0.04	0.12%
	10^2	33.03	33.62	33.39	33.35±0.24	0.72%
	10^3	30.30	30.01	30.26	30.19±0.13	0.43%
	10^4	27.24	27.31	27.03	27.19±0.12	0.44%
	10^5	23.10	23.45	23.53	23.36±0.19	0.80%
	10^6	19.99	19.38	19.86	19.74±0.26	1.33%
	10^7	15.54	15.09	15.32	15.32±0.18	1.20%

SD=Standard Deviation

Table S2. Conformity of detection from co-infection clinical specimens

Results of co-infection	Number of samples	Conformity rate between the two methods
PEDV-G1+RVC	1	100%
PEDV-G2+RVA	5	100%
RVA+RVC	3	100%
PEDV-G2+RVA+RVC	1	100%
